# Validating the Chronic Stress Indicator: A Data-Driven Framework for Integrating Physiological and Socio-Behavioral Measures

**DOI:** 10.1080/15366367.2026.2661304

**Published:** 2026-04-24

**Authors:** Matthew Hill, Emmanuel Obeng-Gyasi, Sayed A. Mostafa

**Affiliations:** a Department of Mathematics & Statistics, North Carolina A&T State University;; b Department of Built Environment, North Carolina A&T State University

**Keywords:** Allostatic load, composite indicators, item weights, MIDUS, uncertainty analysis

## Abstract

Chronic stress contributes to cardiovascular disease, diabetes, and mental health disorders through its cumulative physiological toll, or allostatic load (AL). Building on our previously proposed Chronic Stress Indicator (CSI), this study aims to refine, validate, and compare the CSI using a data-driven framework that integrates physiological, socioeconomic, and behavioral factors. Using data from the MIDUS II biomarker project, a nationally representative sample of U.S. adults aged 34–84, we assessed the performance of the refined CSI and traditional AL indices in predicting multiple stress-related outcomes. Advanced statistical techniques, including the Boruta feature selection algorithm and factor analysis, were applied to optimize biomarker selection and weighting. Sensitivity analyses evaluated the robustness and reliability of each construction. Compared with the original CSI, the refined model incorporating socio-behavioral variables and data-driven weighting demonstrated improved predictive performance for short-term stress outcomes, while both traditional and extended models performed well for long-term outcomes. This work advances the measurement of chronic stress by validating and enhancing the CSI, providing a robust tool for identifying at-risk populations and guiding targeted interventions.

## Introduction

Chronic stress represents a critical public health challenge, manifesting as cumulative physiological dysregulation that elevates risks for cardiovascular diseases, metabolic disorders, immune dysfunction, and mental health disorders (Duric et al., 2016). Despite extensive evidence linking chronic stress to adverse health outcomes, its measurement remains fragmented and inconsistent. The absence of a standardized, reliable measure that can be operationalized using variables commonly available in national health databases limits our ability to identify at-risk populations and design effective interventions (Lazarus, 1990). This lack of a unified approach of measuring stress has been highlighted throughout the literature (Juster et al., 2010). Several studies use varying numbers of sub-indicators which often times come from three or four different biomarker groups, which cause further inconsistencies in composite constructions (Li et al., 2024). Developing a robust, data driven approach to chronic stress measurement is essential because it allows for earlier intervention and more comprehensive analysis on chronic stress.

To address this gap, our prior work (Hill et al., 2024) introduced the Chronic Stress Indicator (CSI) – a data-driven, multidimensional composite that integrates biomarkers and sociodemographic factors to quantify chronic stress more comprehensively than traditional allostatic load (AL) measures. The CSI builds on the concept of allostatic load – the cumulative physiological burden resulting from repeated or chronic stress exposure (McEwen, 1998). Operationalizing AL involves converting the theoretical construct – the cumulative physiological “wear and tear” on the body resulting from chronic stress exposure – into a quantifiable index using measurable biomarkers across multiple biological systems, including cardiovascular, metabolic, and inflammatory domains. These biomarkers are aggregated to reflect the degree of multisystem physiological dysregulation. However, prior approaches have varied considerably in marker selection, weighting, and aggregation methods, leading to inconsistencies across studies.

Building upon our previously proposed CSI framework (Hill et al., 2024), this study focuses on extending and validating that approach through methodological refinement and comparative evaluation. Specifically, we enhance biomarker selection to better represent stress-responsive physiological systems and refine weighting strategies to improve the indicator’s sensitivity and specificity for detecting chronic stress. These refinements are accompanied by a systematic evaluation of the uncertainty introduced at each stage of the construction process. To ensure the indicator’s scalability and practical relevance, we integrate sociodemographic and behavioral factors available in large-scale health surveys, facilitating population-level applications. Multiple data-driven weighting strategies, such as Boruta feature selection and factor analysis, are implemented to assess robustness and interpretability, with the resulting CSI variants benchmarked against established operationalizations of AL. Validation is performed using data from the Midlife in the United States (MIDUS) II biomarker project, a nationally representative dataset that enables comprehensive assessment across multiple stress-related outcomes (Ryff et al., 2025). The CSI is evaluated in relation to both short-term and long-term stress measures, including the Perceived Stress Scale (PSS), Satisfaction with Life Scale (SWLS), and number of lifetime symptoms and chronic conditions (NOLSACC). The first two reflect immediate or subjective stress, whereas the latter capture cumulative life stress and health burden. These validation instruments provide external benchmarks for assessing the CSI’s ability to represent multidimensional stress processes. Testing multiple approaches for CSI construction provides benefits for both data science and public health. From a data science perspective, it enhances robustness, interpretability, and methodological transparency. From a public health standpoint, it supports precise risk stratification and targeted interventions for stress-related health outcomes. Ultimately, this work seeks to advance the standardization and reproducibility of chronic stress measurement, providing a scalable, data-driven framework for both research and practice.

## Materials and methods

### Overview of composite indicator construction

Composite indicators are statistical tools that combine multiple individual measures into a single summary index, allowing for the representation of complex and multidimensional phenomena. They are widely used across disciplines – including economics, social sciences, environmental studies, health, and education – to inform policy and guide data-driven decision-making.

The construction of a composite indicator generally follows several key steps (Nardo et al., 2008). Defining the objective is the initial step of constructing the composite indicator and it involves specifying the dimensions and sub-dimensions to be included to ensure that the indicator adequately represents the underlying concept. Selection of indicators is next, which involves using meaningful variables to capture different dimensions of the construct of interest. It is important to ensure that selected indicators are based on reliable and accessible data sources. Each indicator should align with the theoretical or conceptual framework underpinning the composite indicator. Literature reviews or expert consultations are often used to guide selection.

Data collection and standardization are done immediately following these steps. We must gather data for each selected indicator from credible sources, such as national statistics, international databases, or population-based surveys. Address missing values through appropriate imputation or exclusion, depending on the degree and mechanism of missingness. Since some indicators may be measured on different scales, standardization helps to ensure comparability. Common approaches include min – max scaling, z-score standardized, or dichotomized as shown in Equations (1–3) respectively:

(1) $$X^{\prime }=\frac{X-\text{min}(X)}{\text{max}(X)-\text{min}(X)},$$

(2) $$Z=\frac{X-\mu }{\sigma }$$

(3) $$X=\left\{\begin{array}{c} 1;X\geq \mu \\ 0;X<\mu \end{array}\right.$$

where X is the original value, μ is the mean, and σ is the standard deviation.

Weighting of Indicators comes after standardization and can be done equally or unequally. Equal weighting assigns identical weights to all indicators. This approach is simple but assumes all indicators contribute equally to the construct. Unequal weighting assigns different weights to indicators based on their relative importance. Weighting can be based on expert judgment, policy priorities, or theoretical relevance. Alternatively, weighting can be derived from statistical methods such as Principal Component Analysis (PCA), factor analysis, or the Analytic Hierarchy Process (AHP). PCA, for instance, is often used to extract components that explain the maximum variance in the data and to derive corresponding weights.

Aggregation of the composite involves combining the indicators to form the composite score. This is done through simple linear aggregation or geometric aggregation, which is shown in Equations (4) and (5), respectively:

(4) $$I=\sum_{i=1}^{n}w_{i}X_{i},$$

(5) $$I=\prod_{i=1}^{n}X_{i}^{w_{i}}$$

where $X_{i}$ are the sub-indicators, $w_{i}$ are the corresponding weights, and n is equal to the number of sub-indicators. Next the composite is either, min-max scaled, z-score standardized, or dichotomized as shown in Equations (1–3), respectively. Min-max scaling rescales the composite score to a fixed range (e.g., 0–1 or 0–100) for interoperability. This normalization facilitates comparison across populations, time periods, or datasets.

Validation and sensitivity analysis requires an initial check of the internal consistency of the composite indicator using measures such as Cronbach’s α or Coefficient ω which can be found in Equations (6) and (7):

(6) $$\alpha =\frac{p\overset{‾}{r}}{1+(p-1)\overset{‾}{r}},$$

(7) $$\omega =\frac{{\left(\sum_{p=1}^{p}\lambda_{i}\right)}^{2}}{{\left(\sum_{p=1}^{p}\lambda_{i}\right)}^{2}+\sum_{p=1}^{p}\psi_{i}},$$

where p is the number of sub-indicators, r is the average inter-correlation among them, $\lambda_{i}$ are the factor loadings, and $\psi_{i}$ are the uniqueness’s of the sub-indicators (Nardo et al., 2008; Padilla & Divers, 2016). To test the composite, it is important to evaluate how variations in weighting, normalization, or indicator inclusion affect the composite results. Monte Carlo simulations are frequently employed for this purpose. Assessing the validity of the composite indicator by comparing it with established benchmarks or outcomes theoretically related to the target construct is also critical.

Interpretation and reporting are the final steps and provide a clear explanation of what the composite indicator measures and how to interpret its values. This is done in conjunction with discussing potential limitations, including data quality, methodological assumptions, and sensitivity to weighting or scaling choices. Transparency in reporting strengthens credibility and reproducibility.

### Traditional AL operationalization

The typical operationalization of chronic stress using AL follows the general framework described in (Guidi et al., 2021; Hill & Obeng-Gyasi, 2022; Mauss & Jarczok, 2021). Given a set of biomarkers $X=\left\{x_{1},x_{2},\ldots ,x_{n}\right\}$, AL is defined as in Equation (4), where $w_{1},w_{2},\ldots ,w_{n}$ represent the corresponding weights for the biomarkers, and n denotes the number of sub-indicators. Some studies dichotomize this continuous measure as shown in Equation (3) (Carbone et al., 2022). Alternatively, studies have applied a fixed threshold of three or four for dichotomization (Guidi et al., 2021). In our earlier analyses, we observed that the mean of AL typically falls between 3 and 4, although this range varies across datasets (Hill & Obeng-Gyasi, 2022). While this operationalization is suitable for basic applications, it lacks the precision needed to fully capture chronic stress. Specifically, it focuses exclusively on biological markers and omits the socio-behavioral dimensions that substantially contribute to chronic stress. Moreover, it implicitly assumes equal weighting across all sub-indicators, such that $w_{n}=1\forall n\in \{1,\ldots ,p\}$.

In most cases, the biomarker variables $x_{n}$ are measured on different scales and may not contribute equally to overall AL. Additionally, two individuals can share the same AL value despite having distinct stress profiles. One improvement to this approach involves standardizing or transforming $x_{n}$ prior to aggregation, as expressed in Equation (2). This formulation improves comparability across biomarkers and partially addresses scale heterogeneity. However, our study seeks to further enhance chronic stress measurement through optimized weighting, variable selection, and sensitivity analysisthree elements recognized as essential in composite index construction (Becker et al., 2017; Freudenberg, 2003; Greco et al., 2019; Mariani & Ciommi, 2022).

### Chronic stress indicator

To better understand the multifactorial nature of chronic stress and its influence on disease risk, the CSI integrates sociodemographic factors and physiological biomarkers to produce a continuous stressrisk score, reflecting variation across individuals from low to high chronic stress burden (Hill et al., 2024). This multidimensional design recognizes that stress arises not only from biological dysregulation but also from social and behavioral determinants that shape exposure and resilience (Cohen & Janicki-Deverts, 2012; Folkman & Moskowitz, 2000; Lavretsky & Newhouse, 2012; Rohleder, 2019).

The algorithm in Table 1 summarizes the general procedure for constructing the CSI, following the composite indicator development framework outlined by Nardo et al. (2008). Given a dataset containing both biomarker and socio-behavioral variables X, the algorithm computes CSI values through sequential steps involving data preprocessing, normalization, feature selection, weighting, and aggregation. These refinements enable a more standardized, data-driven measure of chronic stress suitable for population-level research and clinical applications.

To enhance interoperability and facilitate classification, binary versions of both AL and CSI were also created. For each construction, we used statistical quartiles as thresholds to distinguish between low- and high-risk individuals. Sub-indicators in the high-risk quartile were assigned a score of one, and those in the low-risk quartile a score of zero. Summing across sub-indicators yielded an overall AL or CSI score, with individuals scoring greater than or equal to the sample mean classified as high risk and all others as low risk. Using the outlined framework, we generated 32 distinct constructions of AL and CSI, each validated using regression models. One configuration employed an automated feature selection approach based on a penalized factor model that incorporated an adaptive lasso penalty on factor loadings. This procedure effectively shrinks non-informative sub-indicators toward zero, yielding a parsimonious yet interpretable composite score. Additionally, penalized factor models were built for all of the imputed datasets. In 39 of the datasets, total cholesterol and income were removed from CSIAUTO. The remainder of the imputations removed tobacco use, income, and total cholesterol from CSIAUTO.

Table 2 summarizes the biomarker and sociodemographic variables included in the baseline AL and CSI constructions. Additional reconstructions incorporated alternative subsets of these factors to evaluate sensitivity and performance, given known variability in AL computation across studies (Duong et al., 2017). The naming conventions for the AL and CSI constructions are summarized in Table 3.

### Statistical techniques for weighting the CSI

Previous research has emphasized the critical role of weighting in the construction of composite indicators (Greco et al., 2019). The development of the CSI utilized two primary statistical techniques to assign weights. These included Boruta feature selection and Factor Analysis (FA). We applied the Boruta algorithm, a wrapper-based feature selection method in R that uses a random forest classifier, to identify the most important variables for the CSI. The Boruta approach enabled the use of importance scores for weighting of our composites. By comparing the importance of each original feature with that of its randomly permuted shadow counterpart, Boruta effectively ranks biological and sociodemographic variables according to their contribution to chronic stress. Only those features exhibiting statistically significant importance are retained in the CSI. Table 4 shows the Boruta Algorithm. Our target variable for Boruta was DOFOR as the loss of someone has been shown to undermine an individual’s physical and mental health (Umberson et al., 2017).

In parallel, factor analysis was used to derive weights by estimating factor loadings and component scores. These loadings represent the shared variance among observed variables and thus quantify the contribution of each indicator to the latent construct of chronic stress. The detailed factor analysis procedure is provided in Supplementary Table S1. Factor scores were standardized and aggregated to produce the final CSI score.

For comparison, AL was defined following prior research and our earlier work, integrating biomarkers across immune, cardiovascular, and metabolic systems. AL values were constructed through standardization and summation of individual biomarkers, consistent with established approaches in the literature.

### Reliability analysis of CSI

Reliability of the CSI was assessed using Coefficient ω, which has been shown to provide a more accurate estimate of internal consistency than Cronbach’s alpha, particularly when the assumption of tau-equivalence is violated. Since our sub-indicators are not equally weighted, ω is more appropriate for this context. The key assumptions of Coefficient Omega include unidimensionality, heterogeneous factor loadings, and congeneric items.

### Study sample

This study aimed to compare and validate multiple CSIs using data from the MIDUS II Biomarker Project (Ryff et al., 2025). The MIDUS II study is a nationally representative sample of U.S. adults aged 34–84, collected between 2004 and 2009, with an initial sample size of 1,054 participants. It provides extensive biological and sociodemographic data suitable for the operationalization and validation of chronic stress measures. Participants were included if they had complete data for the biomarkers and sociodemographic variables selected for CSI construction and validation. Sampling weights were not applied in this analysis, as only one subsample contained weights, and their use would have substantially reduced the analytic sample. Furthermore, our primary goal was to assess the effect of internal sub-indicator weighting within the CSI framework.

The CSI was constructed using a combination of sociodemographic, behavioral, and biological indicators. Demographic and stress-related factors included age, gender, race/ethnicity, income level, physical activity, education, perceived stress scale (PSS), death of a friend or relative (DOFOR), satisfaction with life scale (SWLS), and the number of lifetime symptoms and chronic conditions (NOLSACC). These measures were obtained through self-administered questionnaires and phone interviews. Health-related behaviors included alcohol consumption and tobacco use.

Laboratory-based biomarkers included root mean square of successive differences (RMSSD), C-reactive protein (CRP), fibrinogen, glycated hemoglobin (HbA1c), high- and low-density lipoprotein cholesterol (HDL, LDL), triglycerides (TG), total cholesterol (TC), albumin (Alb), creatinine clearance (CLCR), body mass index (BMI), waist circumference (WC), systolic and diastolic blood pressure (SBP, DBP), and resting pulse. All blood samples were collected at one of three clinical research centers (University of Wisconsin, Georgetown University, and UCLA), following the standardized MIDUS II biomarker collection protocol (Ryff et al., 2025).

For simplicity, annual income was dichotomized at $22,500—the approximate federal poverty threshold for the study period – coded as one for incomes at or below this value and zero otherwise (Fass, 2009). A full list of variables and their responses are provided in Supplementary Table S2.

### Initial missing data treatment

Missing data were first handled using complete case analysis (CCA), also known as listwise deletion, which excludes participants with missing values in any variable used for analysis. Variables with the highest missingness were tobacco use (38.6%), income (10.1%), and RMSSD (8.73%), while all others had less than 6% missing data. Table 5 shows the percent missing for all variables of interest. After applying CCA, the analytic sample size was n=510. While straightforward to implement, CCA assumes data are missing completely at random (MCAR); violation of this assumption can bias estimates. To assess robustness under the less restrictive missing at random (MAR) assumption, we later conducted sensitivity analyses using multiple imputation via chained equations (MICE).

### Validation framework

Validation of the CSI constructions was performed using three established psychosocial measures: PSS, SWLS, and NOLSACC. The PSS was measured using the 10-item self-rated scale, with responses ranging from 1 (never) to 5 (very often), summed to produce the total PSS score (Cohen et al., 1983; Smith et al., 2014). The SWLS used a 7-point Likert scale, NOLSACC was treated as a continuous variable, and DOFOR was binary (yes/no).

Each CSI and AL configuration was evaluated using regression-based associations with these validation measures. Linear regression models were fitted for PSS, SWLS, and NOLSACC. For each configuration, explanatory power was quantified using $R^{2}$:

$${\text{R}}^{2}=1-\left(\frac{SS_{\text{RES}}}{SS_{\text{TOTAL}}}\right),$$

where $SS_{\text{RES}}$ is the residual sum of squares and $SS_{\text{TOTAL}}$ is the total sum of squares. Configurations were assessed based on their $R^{2}$ values, standardized coefficients (β) and associated p-values, with higher $R^{2}$, |β|, and smaller p-values indicating better model performance. All analyses were conducted in R version 4.4.4 (R Development Core Team, 2022) using the packages Boruta, psych, penfa, and mice. For the Boruta algorithm, we used 500 trees and 100 iterations, which are the default values in R. Future work should look at further optimizing the number of trees and iterations to allow for more stable importance scores. Factor analysis, regression modeling, and sensitivity testing were implemented in R with a significance threshold of 0.05.

## Results

The summary statistics for our studies can be found in Supplementary Table S3. All continuous variables show mean and standard deviation in parentheses, while all categorical variables show proportions. Summary statistics for the MICE datasets were pooled using Rubin’s rule as is customary in the literature (White et al., 2011).

### Reliability results

We evaluated the internal consistency of each AL and CSI construction using Coefficient ω, which captures the degree to which sub-indicators measure a common latent construct, assumed here to represent chronic stress. As shown in Table 6, the CSI AUTO construction – derived via automatic statistical feature selection – achieved the highest reliability (ω=0.45). This was followed by AL10 and CSI10. In contrast, AL5 demonstrated the lowest reliability (ω=0.32), suggesting weaker coherence among its indicators.

### Validation results

**Figure 1.**
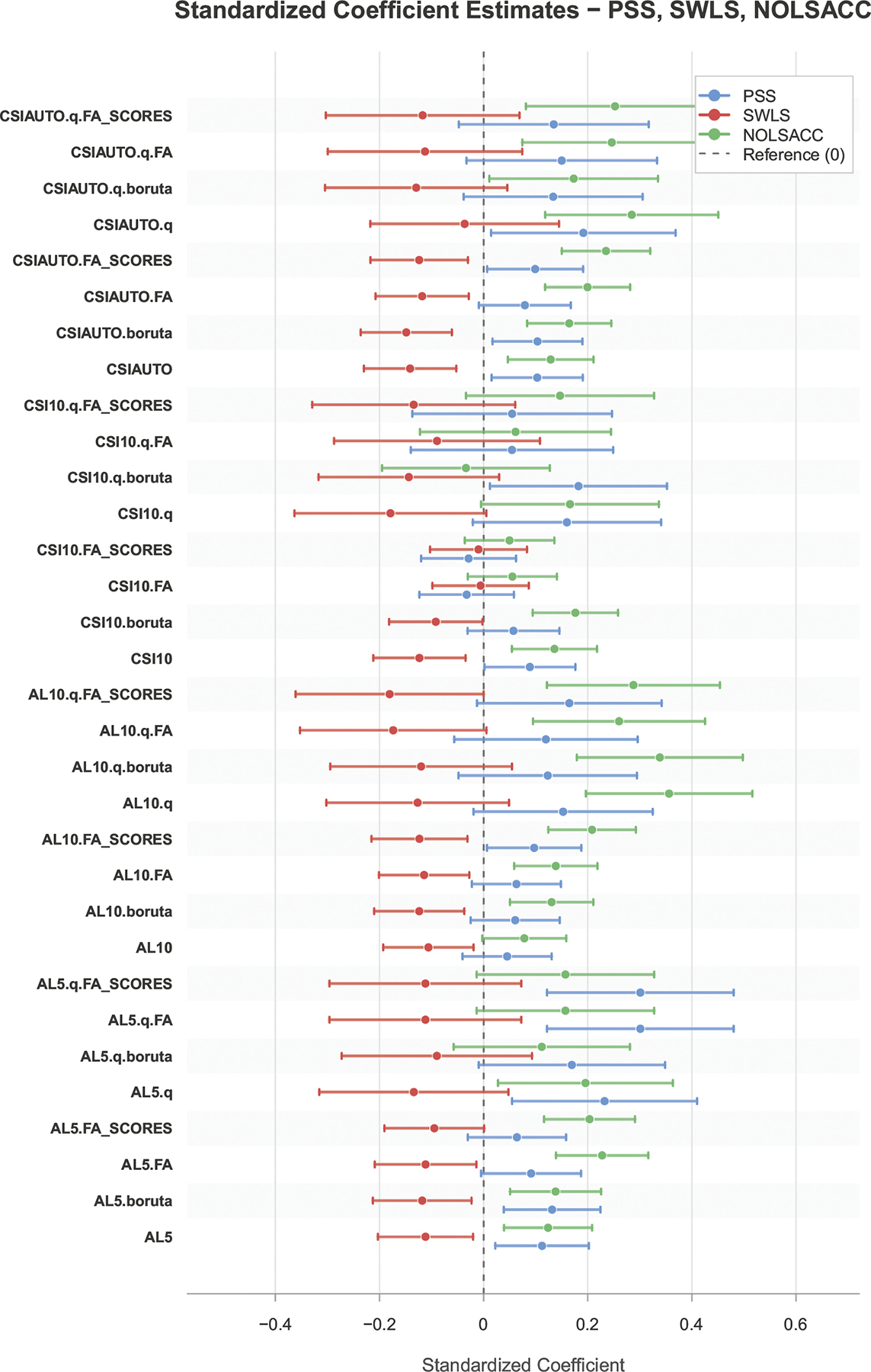
Standardized coefficients and 95% confidence intervals of al and CSI constructions across validation outcomes using CCA data.

**Figure 2.**
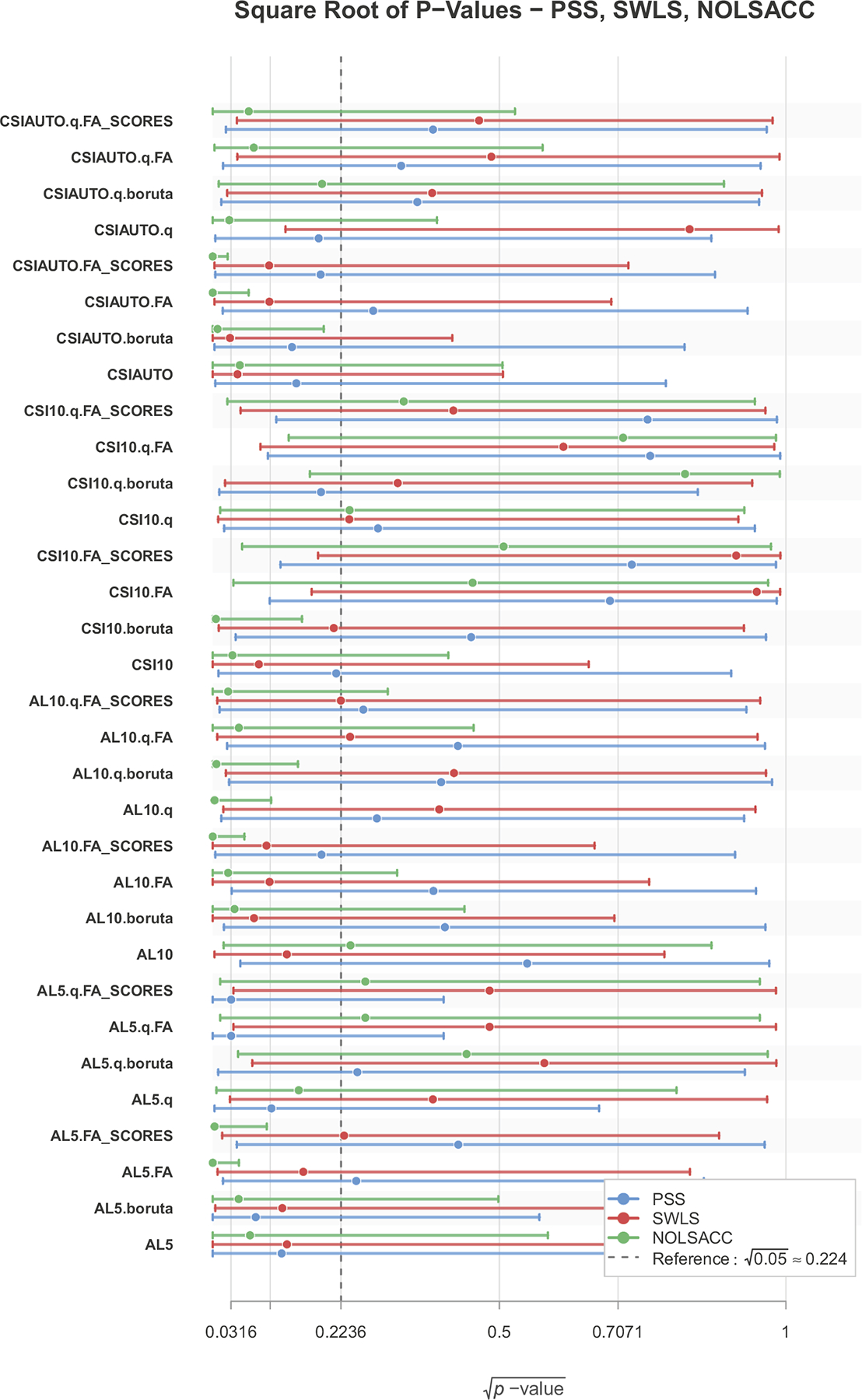
Square root of p-value and 95% confidence intervals of al and CSI constructions across validation outcomes using CCA data.

**Figure 3.**
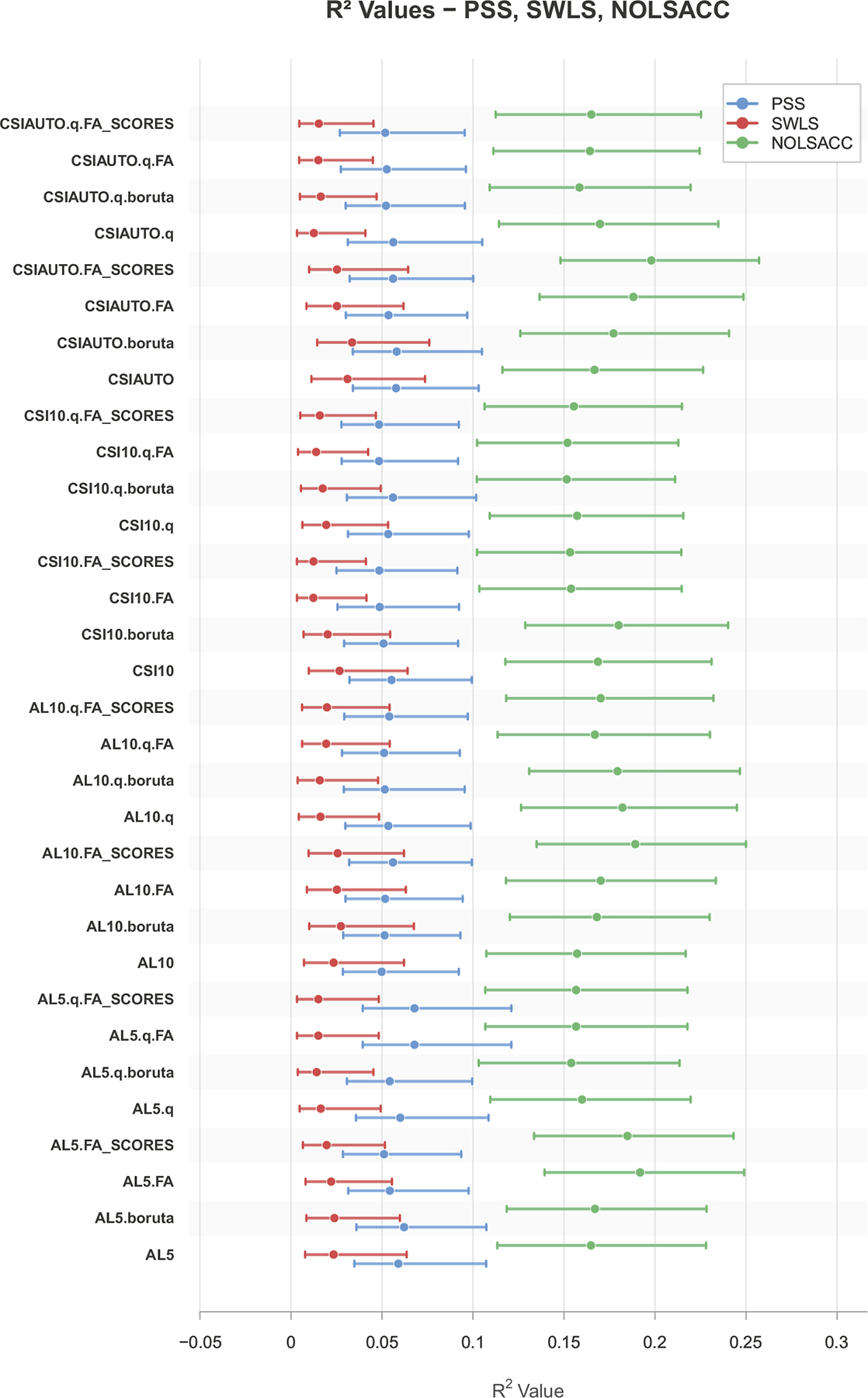
$R^{2}$ values and 95% confidence intervals of al and CSI constructions across validation outcomes using CCA data.

To evaluate predictive performance across different CSI and AL constructions, we fitted linear regression models using multiple validation outcomes. The initial models used complete case analysis (CCA) to handle missing data and did not incorporate survey weights. An alternative approach to handling missing data; namely, multiple imputation, is covered in the uncertainty analysis section below. The predictive validity of each construction was assessed against perceived stress (PSS), subjective well-being (SWLS), and long-term health burden (NOLSACC). All models were adjusted for age and sex. $R^{2}$ and p-value 95% confidence intervals were constructed using 500 bootstrap samples. Figures 1, 2, and 3 show standardized coefficients (β), $\sqrt{p-\text{values}}$, and $R^{2}$ as well as 95% confidence intervals across constructions for PSS, SWLS, and NOLSACC. Constructions are ordered by the number of sub-indicators they include and are labeled to indicate whether variable selection, binary transformation, or statistical weighting was applied. $\sqrt{p-\text{values}}$ were used to aid in the readability of the graphs.

For PSS, the highest-performing models were AL5.q.FA and AL5.q.FA_SCORES (β=0.3009, p-value=0.0010, $R^{2}=0.0679$), which combined five health biomarkers and were dichotomized. AL5.q.FA was weighed via communalities of a one-factor model, while AL5.q.FA.SCORES was constructed using the component scores from a one-factor model.

For SWLS, the top-performing model was CSIAUTO.boruta (β=-0.1486, p-value=0.0009, $R^{2}=0.0336$), which applied penalized factor analysis for variable selection and derived weights from the Boruta algorithm. The second-best model, CSIAUTO (β=-0.1413, p-value=0.0019, $R^{2}=0.0311$), used the same sub-indicators but was equally weighted. Both constructions incorporated variable selection through penalized factor analysis, suggesting that judicious variable selection can improve predictive accuracy. Most models showed negative coefficients for SWLS, which aligns with the inverse relationship between satisfaction and stress.

For NOLSACC, representing long-term health burden, CSIAUTO.FA_SCORES achieved the highest fit (β=0.2349, p-value<0.0001, $R^{2}=0.1980$). This model utilized variable selection and was constructed using component scores. The second-best model, AL5.FA (β=0.2275, p-value<0.0001, $R^{2}=0.1918$), consisted of five health biomarkers and was weighed using communalities from a one-factor model. These models produced the highest standardized coefficients and $R^{2}$ values as well as the lowest p-values. This is expected as the underlying biomarkers are typically used to diagnose many of the symptoms and chronic conditions measured in NOLSACC.

### Uncertainty results

Before finalizing the CSI, we examined the latent structure of the sub-indicators using exploratory factor analysis (EFA). Factor loadings below 0.001 were excluded for clarity (see Supplementary Table S4). Factor 1 was significantly associated with Factor 4 (p-value<0.01), and Factor 2 was correlated with Factor 3. The comparative fit index (CFI = 0.910) and root mean square error of approximation (RMSEA = 0.085) indicated acceptable model fit, supporting the aggregation of sub-indicators into a single composite measure.

We next assessed how sensitive our 32 AL/CSI constructions were to changes in preprocessing and data handling. Specifically, we examined how they shifted under different standardization methods, outlier treatments, and missing-data procedures. This analysis quantifies the robustness of the CSI to methodological choices, ensuring that its interpretation is not unduly influenced by preprocessing artifacts. Two data standardization methods were compared: z-score normalization, which scales variables to have a mean of 0 and a standard deviation of 1, and min – max scaling, which rescales values to a range of [0, 1]. We further assessed the effect of including versus excluding outliers. Our reference data was the data using CCA with z-score standardization.

We also investigated the uncertainty in missing data handling. Specifically, we compared complete case analysis (CCA) with multiple imputation by MICE. For MICE, predictive mean matching (PMM) was used for continuous variables and logistic regression for categorical ones. Following (White et al., 2011), we generated m=40 imputed datasets (since the highest missing value rate in our data was about 38%) with 10 iterations per chain. Parameter estimates were combined using Rubin’s Rules:

$$\tilde{S}=\frac{1}{m}\sum_{i=1}^{m}S_{i},$$

where $S_{i}$ denotes the statistic of interest (e.g., p-value or $R^{2}$) from the $i^{\text{th}}$ imputation.

### Uncertainty in standardization technique

Supplementary Figures S1–S3 show standardized coefficients (β), $\sqrt{p-\text{values}}$, and $R^{2}$ as well as 95% confidence intervals across constructions for PSS, SWLS, and NOLSACC. For these constructions, CCA data was used and all continuous sub-indicators were min-max scaled as opposed to z-score standardization. These conditions produced results that were broadly comparable to Figures 1–3, with the same general hierarchy of outcomes (NOLSACC > PSS > SWLS) and the same pattern of CSI composites outperforming AL composites. However, subtle differences in the absolute magnitude of R^2^ values were noted for specific indicators. FA and FA_SCORES weighted variants within the CSI10 and CSIAUTO families exhibited slightly attenuated R^2^ values relative to the reference, particularly for SWLS. This may reflect the sensitivity of factor-analytically derived weights to the distributional properties of the input variables, which differ between z-score and min-max normalization. The dichotomized indicators showed a similar pattern to the reference, and the AL-based composites remained comparably poor predictors of all three outcomes regardless of standardization approach, reinforcing the robustness of this finding across methodological choices.

### Uncertainty in outlier removal

Next, we investigated the uncertainty in the inclusion/exclusion of outliers. Supplementary Figures S4–S6 show standardized coefficients (β), $\sqrt{p-\text{values}}$, and $R^{2}$ as well as 95% confidence intervals across constructions for PSS, SWLS, and NOLSACC. These conditions closely replicated the reference pattern in the standardized coefficients (β), $\sqrt{p-\text{values}}$, and $R^{2}$ as well as 95% confidence intervals across composite indicators and outcomes. SWLS retained its position as the outcome most strongly explained by CSI-based composites, and the overall range of R^2^ values was consistent with that observed in the reference. The congruence between Supplementary Figures S1–S3 and S4–S6 suggest that outlier removal did not substantively distort the explanatory structure of the composites and that the z-score standardization approach preserves the relative ordering of indicator performance. Minor deviations were observed for NOLSACC, where CSI10.q and CSIAUTO.q variants showed marginally reduced R^2^ compared to the reference, possibly reflecting reduced variability in the outcome after case exclusion.

### Uncertainty in missing data handling

Additionally, we altered the handling of missing data to use MICE as opposed to CCA. Supplementary Figures S7–S9 show standardized coefficients (β), $\sqrt{p-\text{values}}$, and $R^{2}$ as well as 95% confidence intervals across constructions for PSS, SWLS, and NOLSACC. These models used MICE to handle missing data and z-score standardization. These conditions displayed the most notable departures from the reference pattern. Most prominently, the overall scale of R^2^ values was compressed, with the upper bound reduced to approximately 0.20 compared to the 0.30 observed in the reference and complete case analyses. This attenuation is consistent with the well-documented phenomenon of variance inflation in imputed datasets, whereby the incorporation of imputation uncertainty into pooled estimates via Rubin’s Rules tends to moderate point estimates, yielding more conservative assessments of explained variance. These findings also reflect the gain in precision from retaining all participants as opposed to CCA.

Taken together, the analytical conditions demonstrate a high degree of convergence in the performance of composite indicators, lending credibility to the substantive conclusions drawn from any individual approach. The consistent superiority of CSI-based composites, particularly those incorporating social determinants of health alongside physiological biomarkers, across all conditions and outcomes suggests that the broader conceptual scope of the CSI framework is a genuine advantage in predicting psychosocial wellbeing, rather than an artifact of a particular analytical choice. The most consequential methodological difference lies between the complete case analyses and the multiply imputed approach. The compression of R^2^ values underscores the importance of selecting an analytical framework that is appropriate to the inferential goals of the study. Where the primary aim is conservative, population-level inference that accounts for missing data uncertainty, these results should be treated as the most methodologically rigorous. Where the aim is to characterize the maximum explanatory potential of the composites under complete data conditions, the CCA results – and their close alignment with the reference – provide a useful benchmark. The minimal differences between CCA datasets further suggest that the standardization strategy is a secondary methodological concern relative to the choice of imputation approach, and that substantive conclusions regarding composite performance are robust to this choice. Future work may consider whether hybrid approaches, such as applying different aggregation techniques within a multiple imputation framework, yield further improvements in either explanatory power or distributional properties of the pooled estimates. This further reinforces the CSI’s reliability as a robust, data-driven measure of chronic stress.

## Discussion

This study extends the work of Hill et al. (2024), which originally introduced the CSI as a composite tool for comprehensive stress analysis. Building upon that foundation, the present research refines the CSI through methodological enhancements to improve overall validity of the composite. While prior studies – particularly those focusing on AL (Seeman et al., 1997) have centered primarily on physiological biomarkers, they often neglect socio-behavioral stressors that contribute substantially to chronic stress. The refined CSI addresses this limitation by systematically integrating sociodemographic and behavioral variables such as income, education, physical activity, and substance use, thereby operationalizing the biopsychosocial model of health at the population level.

Methodologically, this study advances the CSI framework by employing Boruta feature selection and factor analysis to optimize variable weighting, improving upon earlier summation-based methods by retaining only the most informative predictors (Breiman, 2001). Validation against established measures of stress and well-being (PSS, SWLS) further demonstrates this improved utility relative to traditional AL models. Notably, CSI and AL variants (e.g., CSIAUTO.boruta, AL5.q.FA) achieved superior model fits, highlighting the benefits of using boruta for feature importance and consistent with factor-based approaches observed in previous work (McCaffery et al., 2012). To facilitate adoption and adaptation of the CSI constructions examined in this study, we developed a publicly available R Shiny application that enables users to construct and compare multiple CSI variants using user-supplied data. The application is available at http://mhcode.shinyapps.io/comp.

The CSI offers a comprehensive and flexible tool for assessing chronic stress in both clinical and public health contexts. In healthcare settings, it can aid in identifying individuals at high risk for stress-related conditions, guiding interventions that address both physiological and social determinants of health. In public health, its inclusion of factors like poverty and physical activity enables data-driven planning and equitable resource allocation. Additionally, by leveraging feature selection and factoranalytic weighting, the CSI illustrates how machine learning can enhance composite health measures and improve predictive accuracy. Beyond individual risk assessment, the CSI supports efforts toward health equity by quantifying socioeconomic and behavioral stressors. This allows practitioners and policymakers to advocate for structural interventions such as improving education access, healthcare availability, and neighborhood conditions that reduce chronic stress at the population level.

### Limitations

Despite the promise of the CSI framework studied in this paper, several challenges remain. Reliable computation of the CSI requires comprehensive biomarker and sociodemographic data, which are not uniformly available across healthcare systems. Continuous models, while statistically robust, may be less intuitive for practical interpretation. Moreover, variability in data collection and scaling could hinder cross-population comparability. To address these issues, simplified context-specific CSI variants and practitioner training in composite indicator interpretation are recommended. Integration into electronic health records (EHRs) would further facilitate automated computation and longitudinal tracking.

### Future work

Future research should validate the CSI across diverse populations and expand its coverage to include additional contextual variables such as employment, neighborhood characteristics, and social support. Importantly, extending the CSI to longitudinal data would allow researchers to examine changes in cumulative stress and allostatic load over time, enabling stronger inferences about temporal dynamics and causal pathways. Applying machine learning techniques (e.g., support vector machines, neural networks) may further refine variable selection and weighting. Standardizing AL and CSI methodologies will be essential for comparability and replication. Additionally, this study underscores the critical role of sensitivity analysis in composite indicator construction, which warrants deeper exploration in future longitudinal applications.

## Supplementary Material

Supp 1

Supplemental data for this article can be accessed online at https://doi.org/10.1080/15366367.2026.2661304

## Figures and Tables

**Table 1.** CSI construction algorithm.

**Require**: $X=\left\{x_{1},x_{2},\ldots ,x_{p}\right\}$ Items (e.g., biomarkers and sociodemographic factors)
(1) **Outliers: (Optional)**
**for** each $x_{i}\in X,i\in \{1,2,\ldots ,p\}$, where p is the number of sub-indicators **do**
Compute interquartile range (IQR): $\text{IQR}=Q_{3}-Q_{1}$, Where $Q_{1}$ and $Q_{1}$ are the first and third quartiles respectively Identify potential outliers:
$O_{i}=\left\{\begin{array}{l} x_{i}>Q_{3}+(1.5*\text{IQR})\\ x_{i}<Q_{1}-(1.5*\text{IQR}) \end{array}\right.$
Remove $O_{i}$ from dataset
**end for**
(2) **Standardization:**
a. **Z-Score Standardization**:
$\tilde{X}=\left(\frac{x_{j}-{\bar{x}}_{j}}{\sigma_{x_{j}}}\right)$
where $\tilde{X}$ is the standardized indicators, ${\bar{x}}_{j}$ and $\sigma_{x_{j}}$ are the mean and standard deviation respectively of each biomarker, or
b. **Min-Max Scaling**: $\tilde{X}=\frac{X-\text{min}(X)}{\text{max}(X)-\text{min}(X)}$
(3) **Missing Data Management:**
a. Complete Case Analysis (CCA), or
b. Multivariate Imputation by Chained Equations (MICE)
(4) **Weighting**: Obtain weight matrix $W=\left\{w_{i}\right\}$, through equal or unequal weighting
a. **Equal Weighting**: $W=w_{i}=1\forall i\in \left\{1,2,\ldots ,p\right\}$, or
b. **Unequal Weighting**: See tables 24 & 25 for algorithms of Boruta and factor analysis respectively
(5) **Aggregation:**
a. **Weighted Linear Aggregation (WLA)** $\text{CSI}=\sum_{i=1}^{p}w_{i}{\tilde{x}}_{i}$
(6) **Standardization of CSI:**
$\text{CSI}\prime =\left(\frac{\text{CS}I_{j}-\bar{\text{CSI}}}{\sigma_{\text{CSI}}}\right)$
where CSI′ is the standardized composite, $\bar{\text{CSI}}$ and $\sigma_{\text{CSI}}$ are the mean and standard deviation respectively of the CSI.
(7) **Dichotomization: (Optional)**
$\text{CS}I_{q}=\left\{\begin{array}{l} 1;\text{CSI}\prime \geq \bar{\text{CSI}\prime }\\ 0;\text{CSI}\prime <\bar{\text{CSI}\prime } \end{array}\right.$
**Output**: $\text{CS}I_{q}$ or CSI′

**Table 2.** Variables involved in AL/CSI constructions.

	CSI10	CSIAUTO	AL10	AL5
RMSSD		√		√
SBP	√	√	√	
DBP	√	√	√	√
TC	√		√	
HBA1C	√	√	√	√
BMI	√	√	√	
HDL		√	√	
TG		√	√	
Alb			√	
CLCR		√	√	
CRP		√	√	
LDL		√		√
WC		√		√
Education Level	√			
Poverty status	√			
Alcohol consumption	√	√		
Tobacco use	√	√		
Physical activity	√	√		

**Table 3.** Naming conventions for AL/CSI constructions.

Suffix	Description
.q	Dichotomized using the sample mean
.boruta	Weighted using Boruta algorithm
.FA	Weighted using factor analysis loadings
.FA_SCORES	Used component score as composite

**Table 4.** Boruta algorithm.

Require $\tilde{X}=\left\{{\tilde{x}}_{1},{\tilde{x}}_{2},\ldots ,{\tilde{x}}_{p}\right\}$, Standardized Features from the Algorithm in Table 1.
1. **Initialize**: Mark all features as “Tentative”.
2. **Iterative Comparison** (Repeat until all features are classified or max iterations reached:
a. **Copy & Shuffle:** Create shadow features S by randomly shuffling each tentative feature to generate a baseline distribution.
b. **Train Random Forest Model:** Combine $\tilde{X}$ and S into one feature set.
i. Use DOFOR as the target variable.
ii. Obtain importance scores for both original and shadow features:
$I_{j}=\frac{1}{n_{\text{tree}}}\sum_{t=1}^{n_{\text{tree}}}G_{j}^{t},$
where $G_{j}^{t}$ denotes the decrease in Gini impurity attributed to variable j in tree t.
c. **Compute Threshold and Evaluate Significance**: for each feature j, record a hit if $I_{j}>T$, where $T=\text{max}\left(I_{\text{shadow}}\right)$.
Perform a two-sided binomial test on the cummulative hits.
i. Mark features with significantly higher hits than expected as confirmed or important and keep in the model.
ii. Mark features with significantly lower hits than expected as rejected or unimportant and remove from the model.
iii. Carry remaining tenative or undecided features to the next iteration.
3. **Compute Weights:**
$w_{j}=\frac{{\bar{I}}_{j}}{\sum^{{\bar{I}}_{j}}},$
where ${\bar{I}}_{j}$ is the min-max-scaled mean importance score.
4. **Output**: the weights for all confirmed features: $W=\left\{w_{1},w_{2},\ldots ,w_{k}\right\}$

**Table 5.** Percent missing for variables of this study.

Variable Name	(%) Missing
Tobacco	38.61
Income	10.06
RMSSD	8.73
Alcohol	5.03
Glucose	1.42
CRP	1.33
Fibrinogen	1.33
HBA1C	1.33
HDL	1.04
LDL	1.04
TG	0.85
TC	0.85
Albumin	0.66
Race/Ethnicity	0.28
Education	0.28
PSS	0.28
SWLS	0.28
CLCR	0.28
Pulse	0.19
BMI	0.09
WC	0.09
SBP	0.09
DBP	0.09
Age	0
Sex	0
Physical Activity	0
DOFOR	0
NOLSACC	0

**Table 6.** Coefficient Omega for AL/CSI constructions.

Construction	CSI AUTO	AL10	CSI10	AL5
Omega (ω)	0.45	0.34	0.33	0.32
