# Supplementary material for: Validating the Chronic Stress Indicator: A Data-Driven Framework for Integrating Physiological and Socio-Behavioral Measures
Source: Measurement ( Mahwah N J). Author manuscript; Available in PMC 2026 Jun 16. (PMC13267912; doi:10.1080/15366367.2026.2661304)
Supplement: Supp 1 [file NIHMS2171328-supplement-Supp_1.pdf]

# Validating the Chronic Stress Indicator: A Data-Driven Framework for Integrating Physiological and Socio-Behavioral Measures

## Supplementary Material

This document provides technical details and expanded data summaries supporting the main manuscript. Table S1 outlines the specific factor analysis algorithm and the Maximum Likelihood Estimation (MLE) process used for latent factor modeling and score computation. Table S2 provides a comprehensive data dictionary for the MIDUS variables, including response levels and measurement units. Table S3 offers a comparative look at the summary statistics for the Complete Case Analysis (CCA) and Multiple Imputation by Chained Equations (MICE) datasets, while Table S4 presents the standardized factor loadings for the sub-indicators across four distinct factors. Finally, the uncertainty analysis results (Figures S1–S9) illustrate the robustness of the Allostatic Load (AL) and Cumulative Stress Index (CSI) constructions, assessing standardized coefficients, p-values, and $R^2$ values across various sensitivity checks, including min-max scaling, outlier removal, and z-score standardization.

Table S1: The factor analysis algorithm used.

| |
|---|
| **Require:** $\tilde{X} = \{\tilde{x}_1, \tilde{x}_2, \dots, \tilde{x}_p\}$, Standardized Features from Algorithm 1 |
| **Define Factor Model:**<br>$$O = \mu + LF + \epsilon$$<br>Where $O$ is the observed variables, $\mu$ is the mean vector, $L$ is the factor loadings matrix, $F$ is the latent factors and $\epsilon$ is the residual error<br>**Covariance Structure:**<br>$$\Sigma = LL^T + \Psi$$<br>Where $\Sigma$ is the model-implied covariance matrix and $\Psi$ is the diagonal matrix of unique variances |
| **Set Initial Estimates:**<br>$$L^{(0)} \ and \ \Psi^{(0)}$$<br>Compute sample covariance matrix $S$ from $X$ |
| **Maximum Likelihood Estimation (Expectation-Maximation Algorithm):**<br>Mean of factors given data:<br>$$E(F\|X) = (L^T\Psi^{-1}L + I)^{-1}L^T\Psi^{-1}(X - \mu)$$<br>Expected factor covariance:<br>$$E(FF^T\|X) = (L^T\Psi^{-1}L + I)^{-1} + E(F\|X)E(F\|X)^T$$ |

| **Update estimates of $L$ and $\Psi$ and check for convergence:** $$logL(\theta) = -\frac{1}{2}(p * log(2\pi) + log\|\Sigma\| + tr(S\Sigma^{-1}))$$ If $\Delta\ logL(\theta) < threshold$, stop iteration |
|---|
| **Compute Factor Scores (Optional):** $$\hat{F} = WX$$ $W$ is the weight matrix obtained from: $$W = (\Sigma^{-1}L(L^T\Sigma^{-1}L)^{-1})^2$$ |
| **Output:** $\hat{F}$ or $L$ |

Table *S2*: MIDUS data variables response levels.

| Variable Name | Variable Responses |
|---|---|
| Sex | Male,Female |
| Age | Positive Integer |
| Income | less than \$0, \$0 (NONE), (\$1 - \$1,999), (\$2,000 - \$3,999), (\$4,000 - \$5,999), (\$6,000 - \$7,999), (\$8,000 - \$9,999), (\$10,000 - \$11,999), (\$12,000 - \$13,999), (\$14,000 - \$15,999), (\$16,000 - \$17,999), (\$18,000 - \$19,999), (\$20,000 - \$22,499), (\$22,500 - \$24,999), (\$25,000 - \$27,499), (\$27,500 - \$29,999), (\$30,000 - \$32,499), (\$32,500 - \$34,999), (\$35,000 - \$37,499), (\$37,500 - \$39,999), (\$40,000 - \$42,499), (\$42,500 - \$44,999), (\$45,000 - \$47,499), (\$47,500 - \$49,999), (\$50,000 - \$54,999), (\$55,000 - \$59,999), (\$60,000 - \$64,999), (\$65,000 - \$69,999), (\$70,000 - \$74,999), (\$75,000 - \$79,999), (\$80,000 - \$84,999), (\$85,000 - \$89,999), (\$90,000 - \$94,999), (\$95,000 - \$99,999), (\$100,000 - \$109,999), (\$110,000 - \$119,999), (\$120,000 - \$129,999), (\$130,000 - \$139,999), (\$140,000 - \$149,999), (\$150,000 - \$174,999), (\$175,000 - \$199,999), and \$200,000 or more |
| Education | "no school/some grade school (1-6)", "eighth grade/junior high school (7-8)", "some high school (9-12 no diploma/no General Educational Development (GED))", "GED", "graduated from high school", "1 to 2 years of college, no degree yet", "3 or more years of college, no degree yet", "graduated from 2 year college, vocational school, or associates degree", "graduated from 4 or 5 year college, or bachelor's degree", "some graduate school", "master's degree", and "PH.D., ED.D, MD, DDS, LLB, LLD, JD, or other professional degree" |
| Race/Ethnicity | "White", "Black and/or African American", "Asian", "Native American or Alaska Native Aleutian Islander/Eskimo", "Native Hawaiian or Pacific Islander" and "Other (Specify)" |
| Alcohol Consumption | Yes,No |
| Regularly Use Tobacco Now or in the Past | Yes,No |
| Physical Activity | Yes,No |

| | |
|---|---|
| Perceived Stress Scale (PSS) | Positive Integer |
| Death of Friend or Relative (DOFOR) | Yes,No |
| Satisfaction with Life Scale (SWLS) | Positive Integer |
| Number of Lifetime Symptoms and Chronic Conditions (NOLSACC) | Positive Integer |
| Systolic Blood Pressure (SBP) | Positive Integer |
| Diastolic blood pressure (DBP) | Positive Integer |
| Total cholesterol (TC) levels (mg/dL) | Positive Integer |
| High-density lipoprotein (HDL) cholesterol (mg/dL) | Positive Integer |
| Low-density lipoprotein (LDL) cholesterol (mg/dL) | Positive Integer |
| Glycosylated hemoglobin (HbA1c) (%) | Positive Integer |
| Albumin (Alb) (mg/dL) | Positive Decimal |
| Triglyceride (TG) (mg/dL) | Positive Integer |
| Body mass index (BMI) | Positive Decimal |
| Creatinine clearance (CLCR) (mg/dL) | Positive Decimal |
| C-reactive protein (CRP) (μg/ml) | Positive Decimal |
| Fasting glucose (mg/dL) | Positive Decimal |
| Fibrinogen (mg/dL) | Positive Integer |
| Pulse | Positive Integer |
| Waist Circumference (WC) (cm) | Positive Integer |

Table *S3*: Summary statistics comparing the CCA and MICE datasets.

| | **CCA** | **MICE** |
|---|---|---|
| **Characteristic** | **Mean(sd)/Proportion** | **Mean(sd)/Proportion** |
| Age | 52.70 (10.96) | 55.26 (11.78) |
| Race/Ethnicity | | |
| White | 91% | 93.04% |
| Black | 4.6% | 2.59% |
| Other | 4.3% | 4.08% |
| Asian | 0.1% | 0.29% |
| Sex | | |
| Male | 44% | 45.26% |
| Female | 56% | 54.74% |
| Education | | |
| No School/Some Grade School | 0.2% | 0.01% |
| Eighth Grade/Junior High School | 0.5% | 0.78% |
| Some High School | 2.2% | 2.67% |

| | | |
|---|---|---|
| GED | 0.5% | 0.77% |
| Graduated From High School | 18% | 19.90% |
| 1 To 2 Years Of College, No Degree Yet | 15% | 17.22% |
| 3 Or More Years Of College, No Degree Yet | 4.6% | 4.10% |
| Grad. From 2-Year College, Vocational School, Or Assoc. Deg. | 6.9% | 7.89% |
| Graduated From A 4- Or 5-Year College, Or Bachelor's Deg. | 28% | 22.90% |
| Some Graduate School | 4.0% | 4.57% |
| Master's Degree | 15% | 14.64% |
| Ph.D., Ed.D., Md, Dds, Llb, Lld, Jd, Or Other Profess'nl Deg. | 4.6% | 4.46% |
| Income (At or above poverty line) | | |
| No | 54% | 54.23% |
| Yes | 46% | 45.77% |
| Alcohol Consumption | | |
| No | 29% | 67.32% |
| Yes | 71% | 32.68% |
| Perceived Stress Scale (PSS) | 21.71 (6.28) | 21.70 (6.19) |
| Satisfaction With Life Scale (SWLS) | 4.92 (1.32) | 4.90 (1.28) |
| Number of Lifetime Symptoms & Chronic Conditions (NOLSACC) | 3.59 (2.66) | 3.80 (2.77) |
| Physical Activity | | |
| No | 19% | 78.84% |
| Yes | 81% | 21.16% |
| Death of Friend or Relative (DOFOR) | | |
| No | 44% | 49.72% |
| Yes | 56% | 50.28% |
| Regular Use Tobacco Now Or In The Past | | |
| No | 53% | 46.15% |
| Yes | 47% | 53.85% |
| BMI (Body Mass Index) | 28.87 (5.47) | 29.18 (6.01) |
| Urine Creatinine (mg/dL) | 76.70 (49.09) | 77.38 (50.04) |
| Blood Triglycerides (mg/dL) | 129.27 (85.31) | 135.46 (139.20) |
| Blood C-Reactive Protein (ug/mL) | 2.49 (3.52) | 2.73 (4.34) |
| RMSSD (ms) | 22.76 (0.81) | |
| DBP | 74.87 (10.03) | 75.04 (10.25) |
| SBP | 129.10 (16.95) | 131.11 (17.68) |
| HBA1C (%) | 5.90 (0.80) | 5.99 (0.91) |
| LDL (mg/dL) | 104.59 (36.37) | 106.22 (35.40) |
| HDL (mg/dL) | 55.79 (18.36) | 54.58 (17.60) |
| Waist Circumference (cm) | 96.20 (17.42) | 96.76 (16.57) |

| | | |
|---|---|---|
| Urine Albumin (mg/dL) | 0.61 (2.89) | 0.64 (3.32) |
| TC (mg/dL) | 185.74 (40.95) | 187.17 (40.09) |

Table S4: Standardized factor loadings (*significant at the 1% level).

| Sub Indicators | Factor 1 | Factor 2 | Factor 3 | Factor 4 |
|---|---|---|---|---|
| RMSSD | | 0.082 | -0.122* | -0.138* |
| SBP | -0.037 | 0.124* | 0.017 | 0.262* |
| DBP | 0.115 | 0.046 | 0.146* | 0.136* |
| TC | -0.005* | 0.302* | 0.891* | 0.001 |
| HBA1C | 0.019 | 0.051 | -0.091 | 0.190* |
| HDL | 0.984* | -0.012 | | |
| TG | 0.583 | 0.841* | | |
| ALB | 0.052 | -0.030 | 0.025 | -0.049 |
| CLCR | -0.247* | 0.022 | 0.031 | 0.083 |
| BMI | 0.002 | | -0.005 | 0.906* |
| CRP | -0.041 | -0.038 | 0.041 | 0.375* |
| LDL | 0.247 | -0.003 | 1.004* | -0.002 |
| WC | 0.126* | 0.002 | -0.004 | 0.770* |
| EDUCATION | -0.004 | 0.020 | 0.087 | 0.048 |
| INCOME | -0.116 | 0.046 | -0.048 | 0.027 |
| ALCOHOL | -0.087 | -0.005 | 0.066 | -0.069 |
| TOBACCO | 0.004 | | -0.025 | 0.071 |
| PHYSICAL ACTIVITY | 0.065 | -0.072 | -0.036 | 0.207* |

## Uncertainty Analysis Results

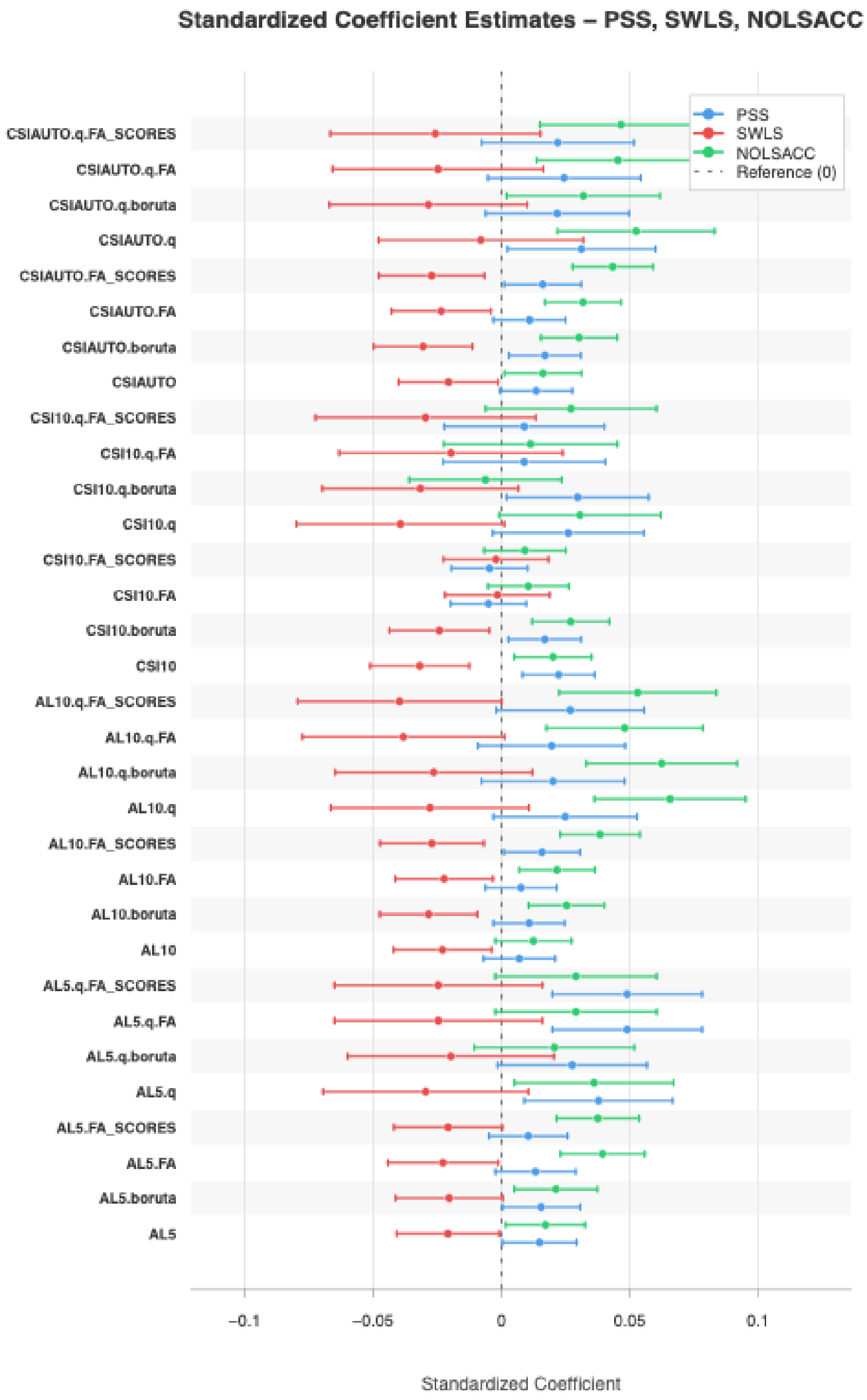


Figure S1: Standardized Coefficients and 95% confidence intervals of AL and CSI constructions across validation outcomes using CCA data with min-max scaling.

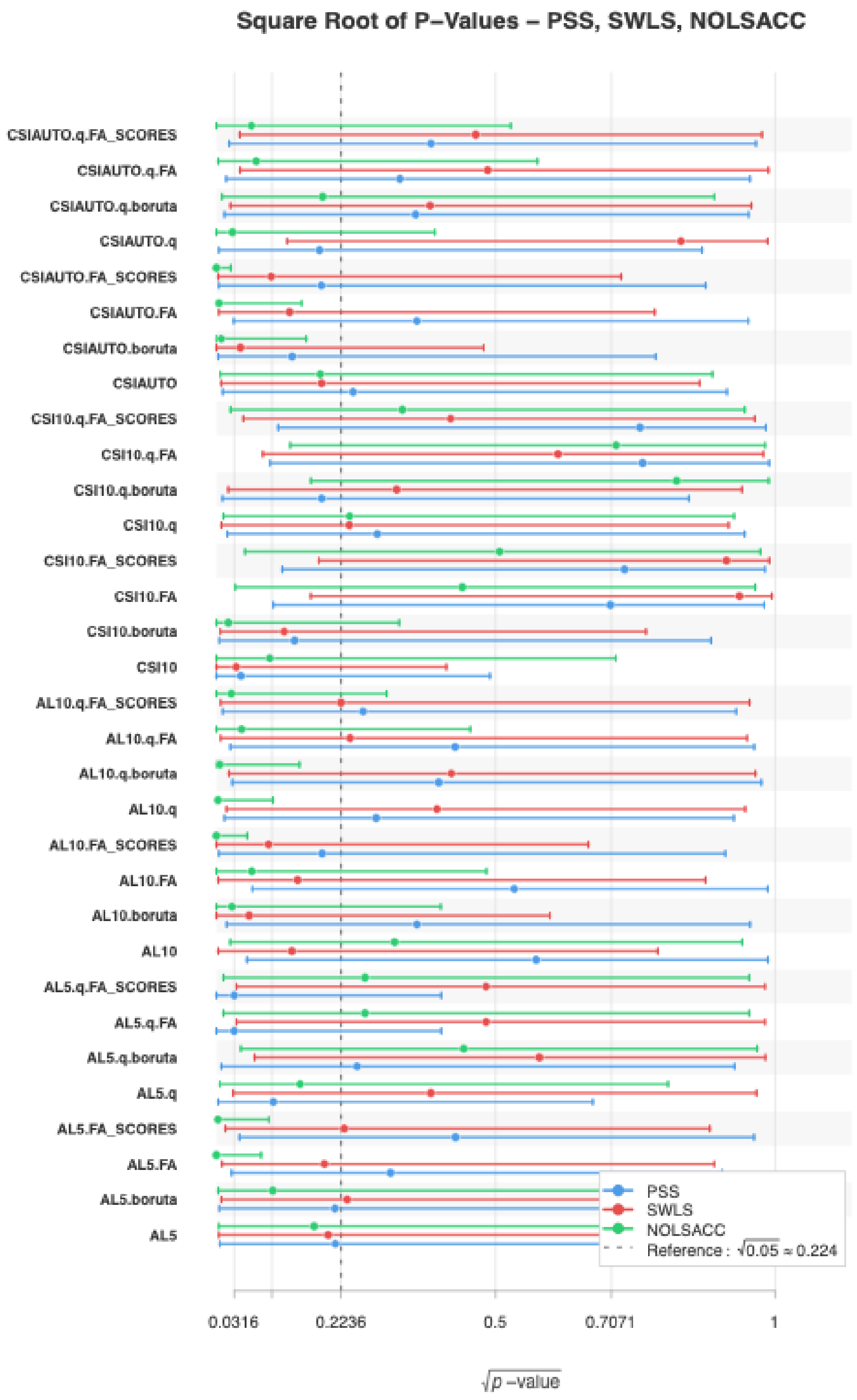


Figure S2: Square root of p-value and 95% confidence intervals of AL and CSI constructions across validation outcomes using CCA data with min-max scaling.

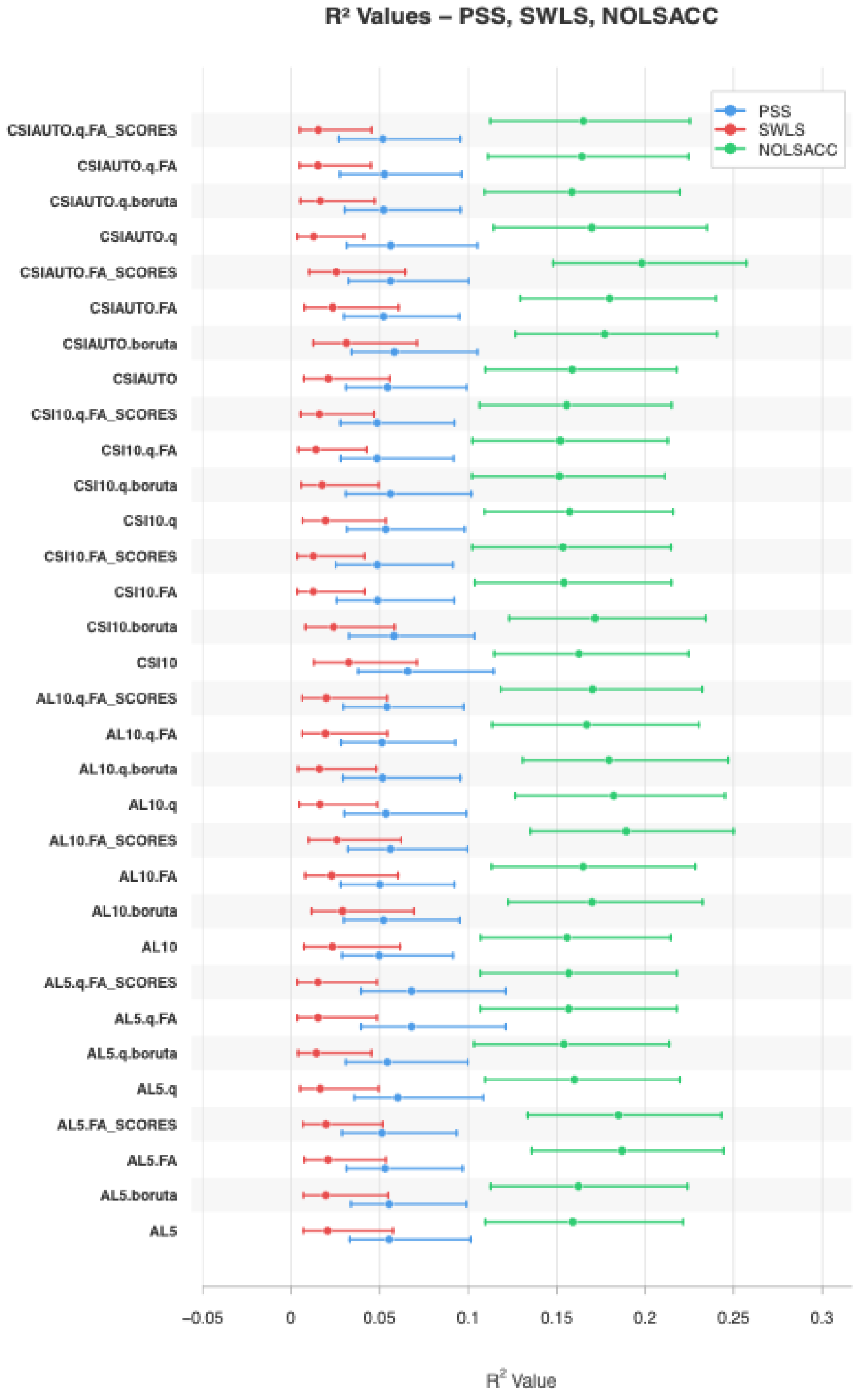


Figure S3: $R^2$ and 95% confidence intervals of AL and CSI constructions across validation outcomes using CCA data with min-max scaling.

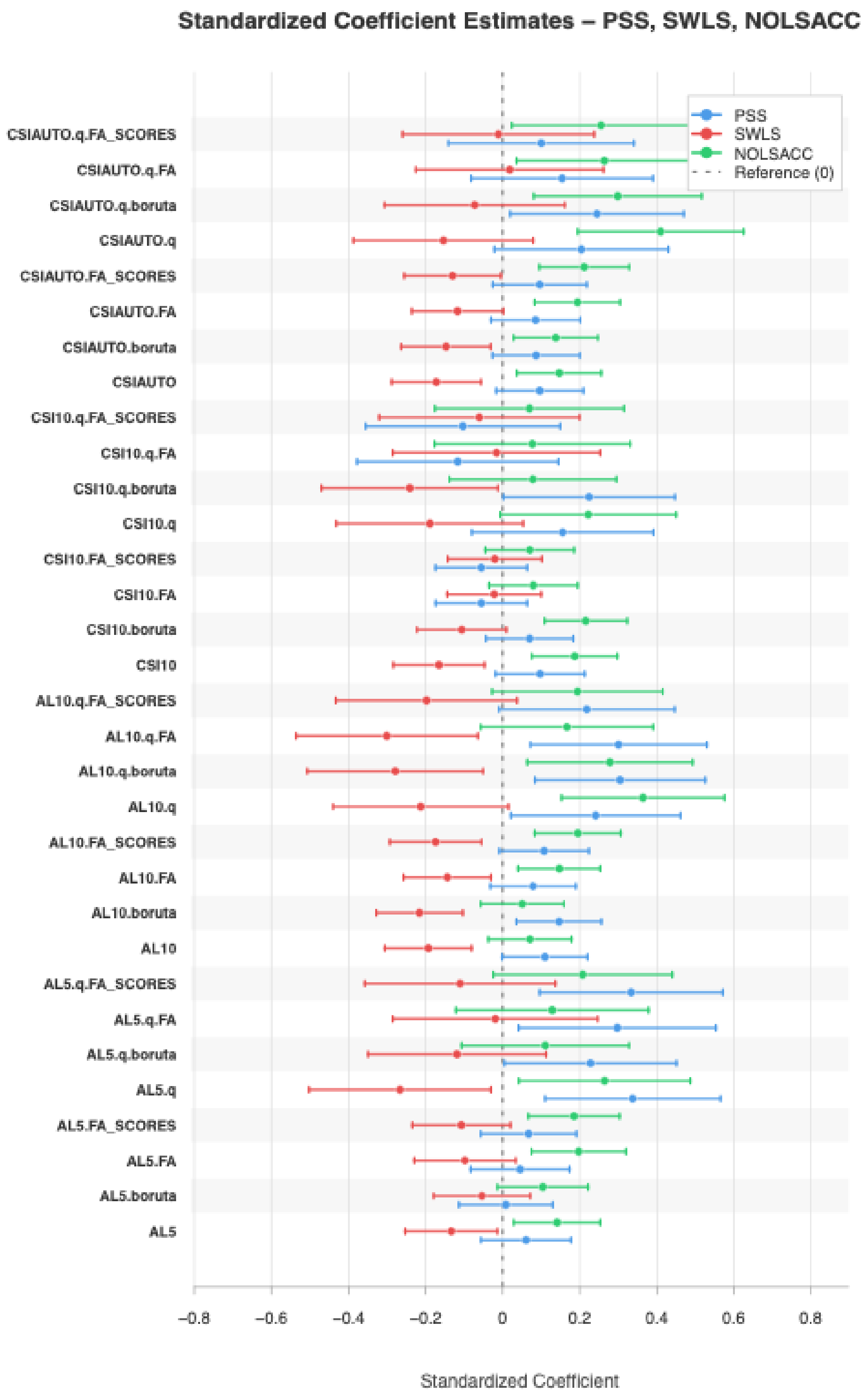


Figure “S4”: Standardized Coefficients and 95% confidence intervals of AL and CSI constructions across validation outcomes using CCA data with outliers removed.

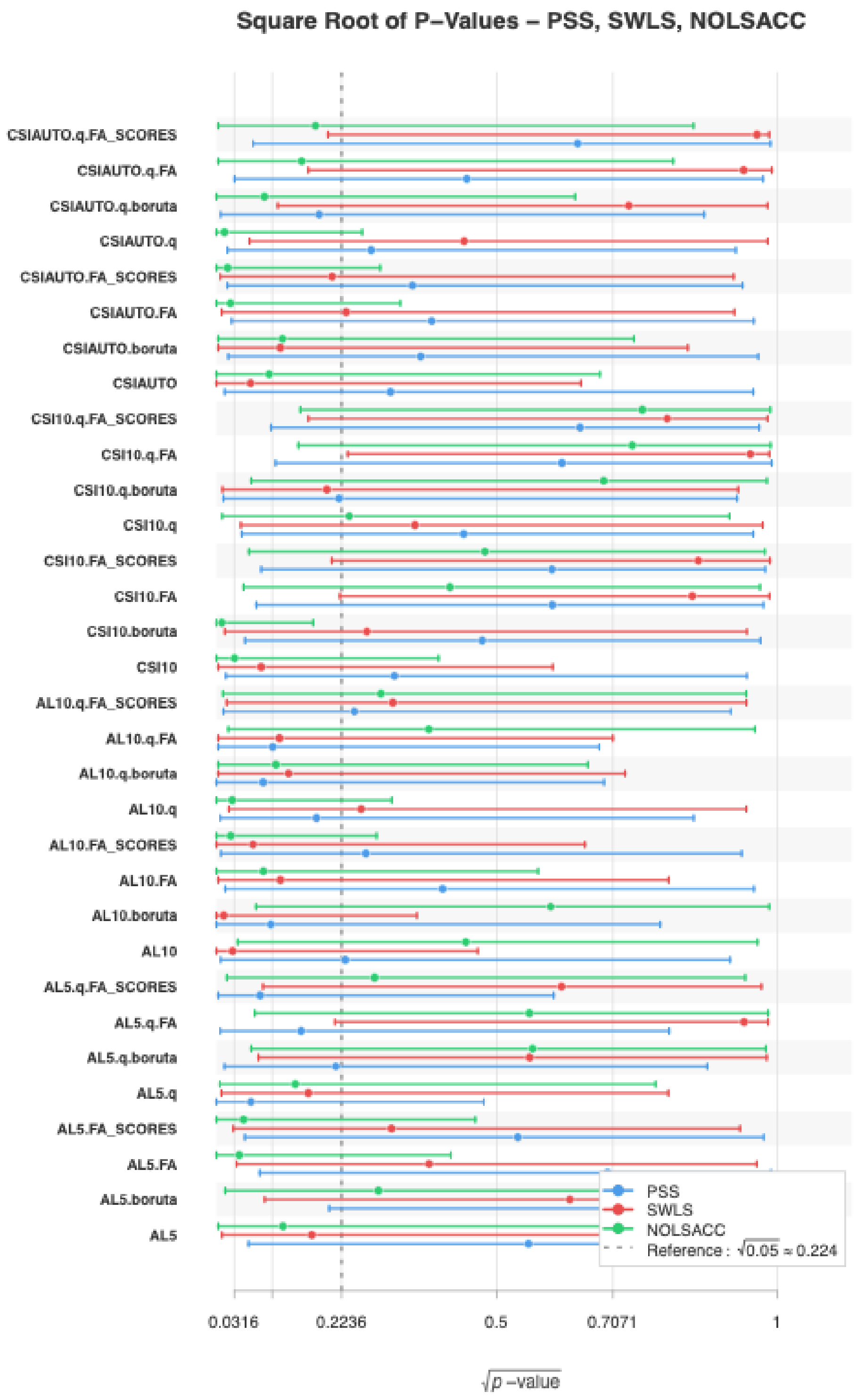


Figure “S5”: Square root of p-value and 95% confidence intervals of AL and CSI constructions across validation outcomes using CCA data with outliers removed.

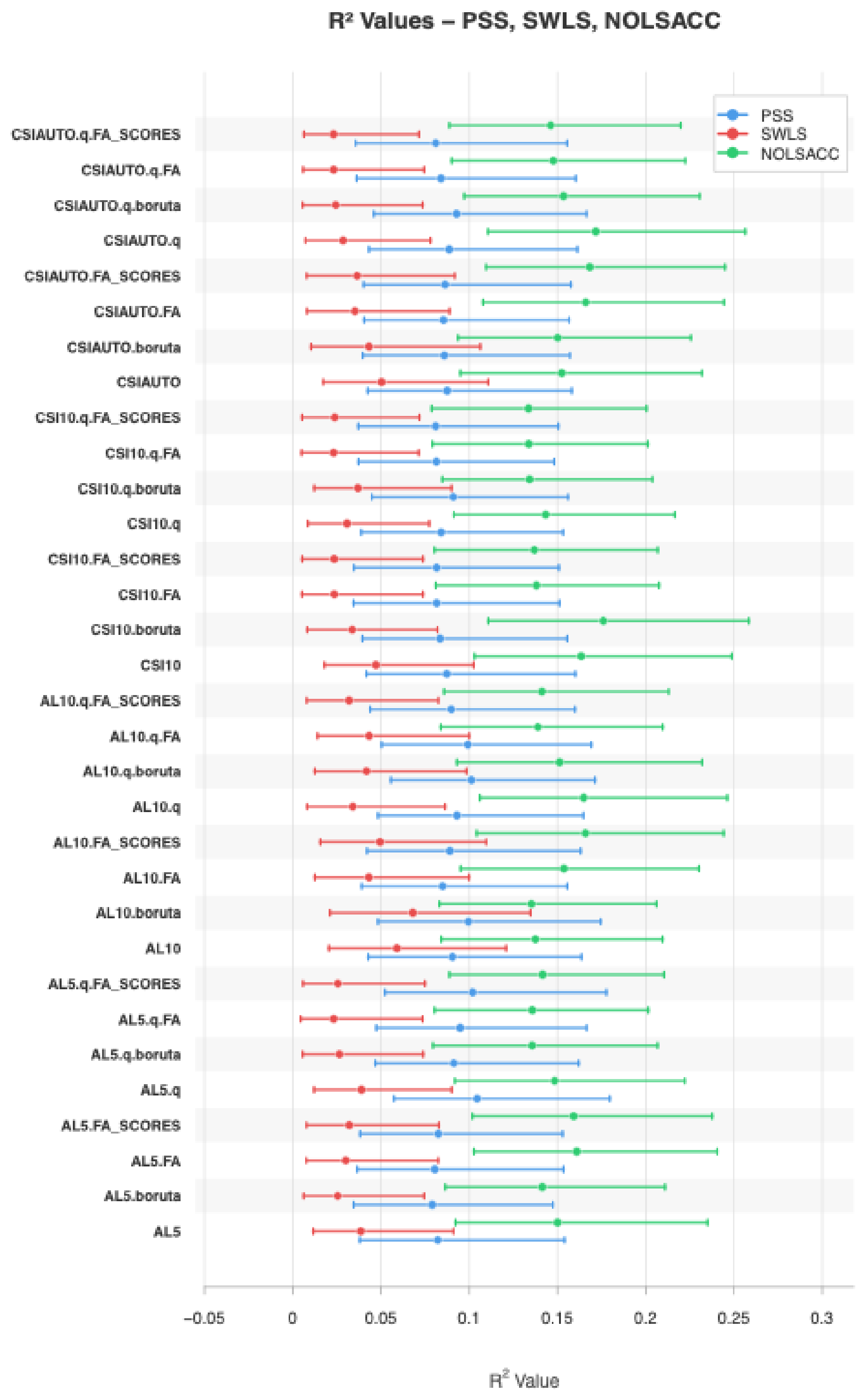


Figure "S6": $R^2$ and 95% confidence intervals of AL and CSI constructions across validation outcomes using CCA data with outliers removed.

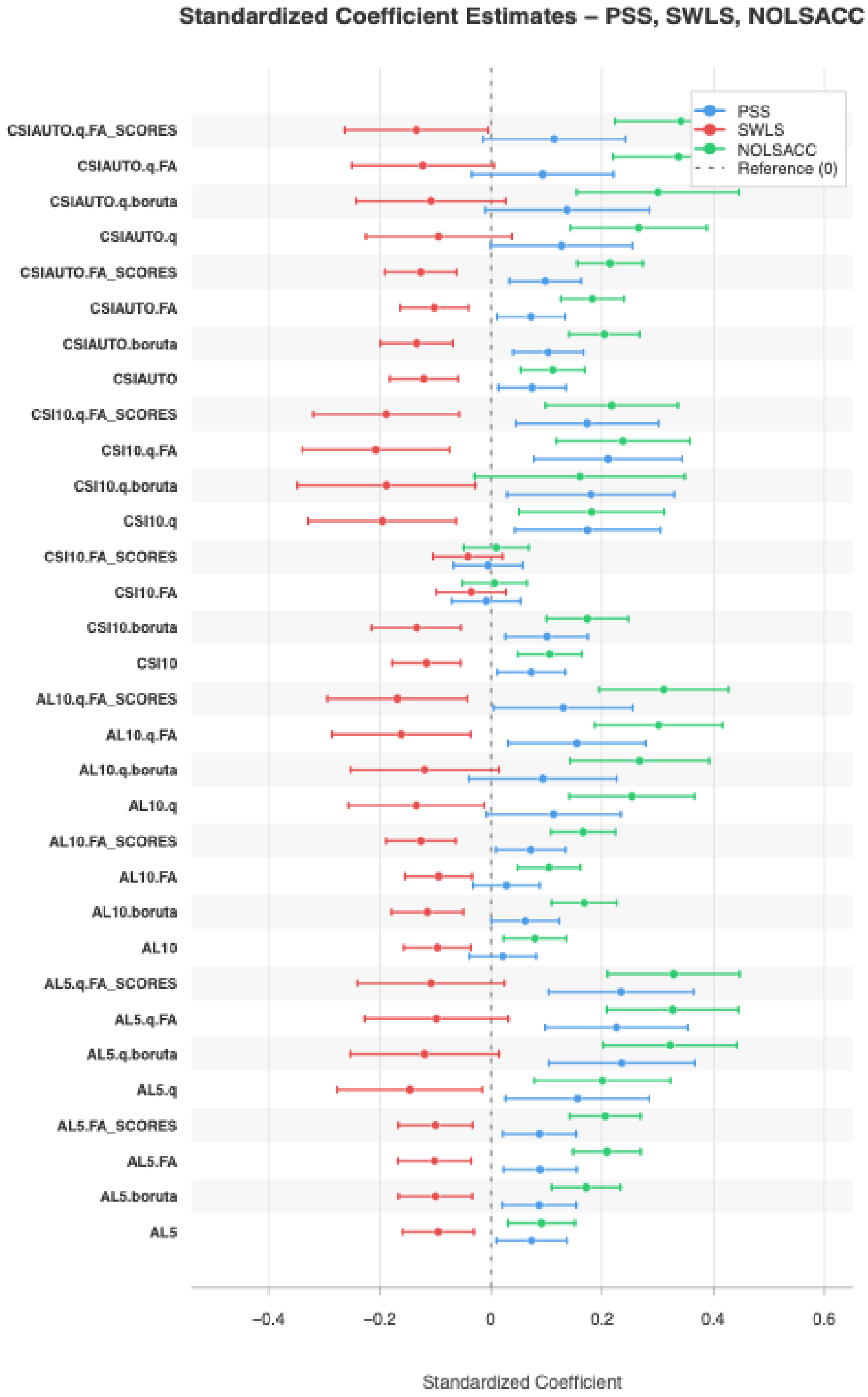


Figure “S7”: Standardized Coefficients and 95% confidence intervals of AL and CSI constructions across validation outcomes using MICE data with z-score standardization.

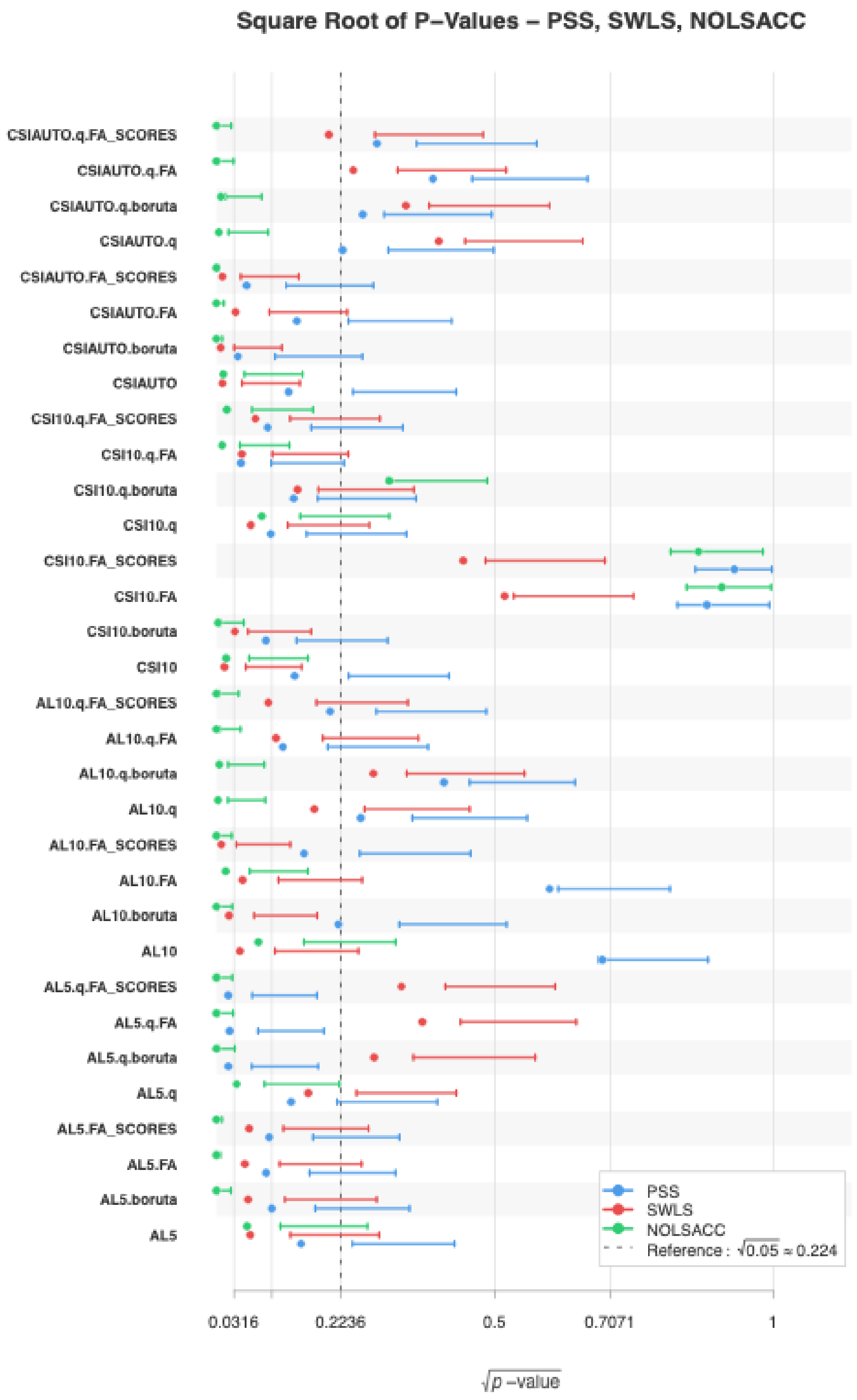


Figure “S8”: Square root of p-value and 95% confidence intervals of AL and CSI constructions across validation outcomes using MICE data with z-score standardization.

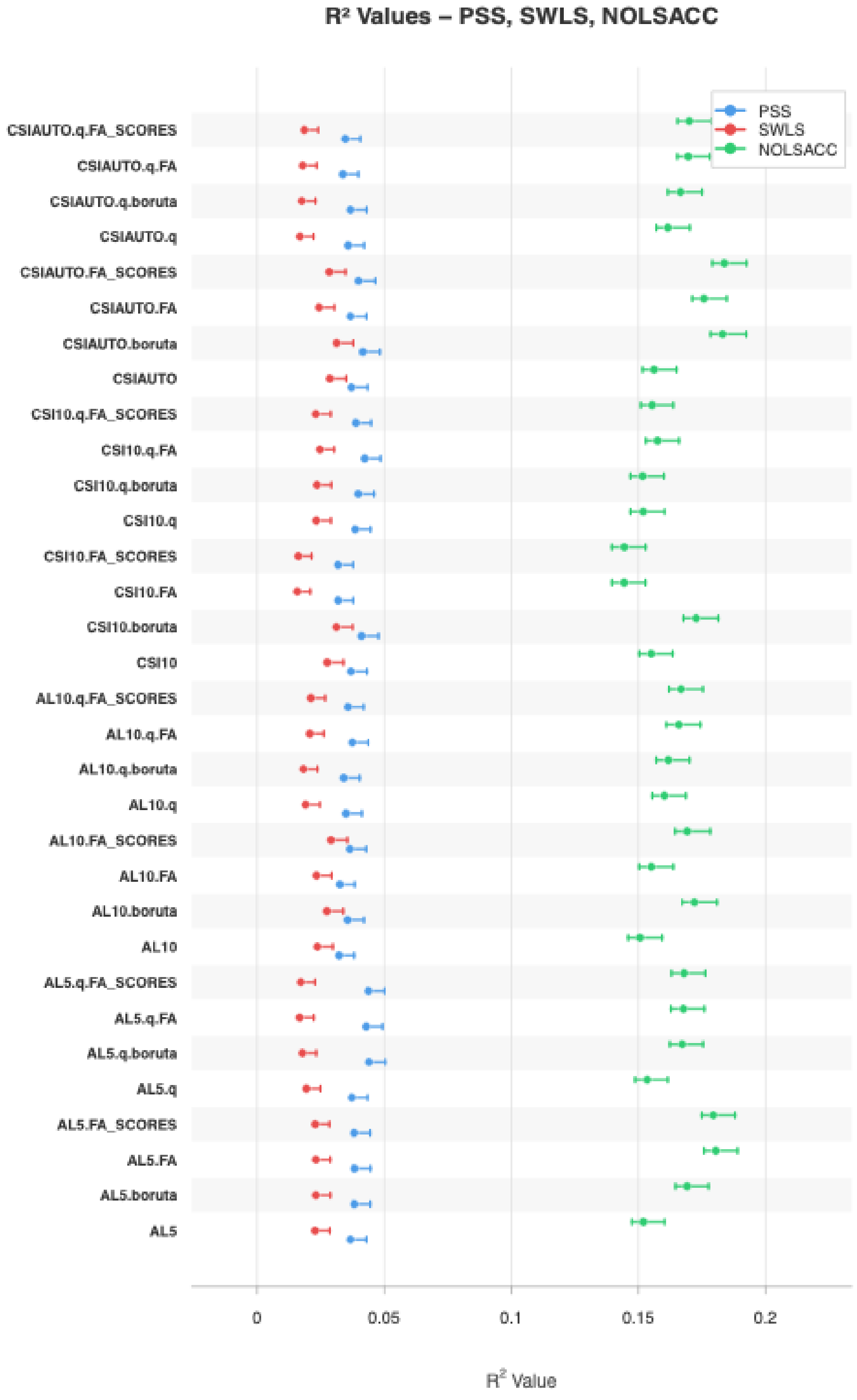


Figure “S9”: $R^2$ and 95% confidence intervals of AL and CSI constructions across validation outcomes using MICE data with z-score standardization.
